# The Endocannabinoid System, Aggression, and the Violence of Synthetic Cannabinoid Use, Borderline Personality Disorder, Antisocial Personality Disorder, and Other Psychiatric Disorders

**DOI:** 10.3389/fnbeh.2018.00041

**Published:** 2018-03-27

**Authors:** Nathan J. Kolla, Achal Mishra

**Affiliations:** ^1^Department of Forensic Psychiatry, Centre for Addiction and Mental Health, Toronto, ON, Canada; ^2^Department of Psychiatry, Faculty of Medicine, University of Toronto, Toronto, ON, Canada; ^3^Waypoint Centre for Mental Health Care, Waypoint Research Institute, Penetanguishene, ON, Canada

**Keywords:** endocannabinoid system, aggression, synthetic cannabinoids, borderline personality disorder, antisocial personality disorder

## Abstract

Endogenous and exogenous cannabinoids bind to central cannabinoid receptors to control a multitude of behavioral functions, including aggression. The first main objective of this review is to dissect components of the endocannabinoid system, including cannabinoid 1 and cannabinoid 2 receptors; the endogenous cannabinoids anandamide and 2-arachidonoylglycerol; and the indirect cannabinoid modulators fatty acid amide hydrolase and monoacylglycerol lipase; that have shown abnormalities in basic research studies investigating mechanisms of aggression. While most human research has concluded that the active ingredient of marijuana, Δ9-tetrahydrocannabinol, tends to dampen rather than provoke aggression in acute doses, recent evidence supports a relationship between the ingestion of synthetic cannabinoids and emergence of violent or aggressive behavior. Thus, another objective is to evaluate the emerging clinical data. This paper also discusses the relationship between prenatal and perinatal exposure to cannabis as well as use of cannabis in adolescence on aggressive outcomes. A final objective of the paper is to discuss endocannabinoid abnormalities in psychotic and affective disorders, as well as clinically aggressive populations, such as borderline personality disorder and antisocial personality disorder. With regard to the former condition, decreased anandamide metabolites have been reported in the cerebrospinal fluid, while some preliminary evidence suggests that fatty acid amide hydrolase genetic polymorphisms are linked to antisocial personality disorder and impulsive-antisocial psychopathic traits. To summarize, this paper will draw upon basic and clinical research to explain how the endocannabinoid system may contribute to the genesis of aggressive behavior.

## Introduction

Aggression is a multifaceted behavior that leads to harm toward the self or others and whose genesis can be traced to a multiplicity of individual and environmental factors. Essential to understanding mechanisms of aggression or violence (we use these terms interchangeably for the purpose of this review) is a thorough dissection of relevant neurobiological systems. Here, we focus on the neurochemistry of exogenous cannabinoids and features of endocannabinoid system (ECS) signaling that relate to aggressive behavior. This article is divided into five sections. First, we discuss the animal literature probing the ECS and aggression. Second, we discuss developmental effects of cannabis use during prenatal and perinatal periods as well as in adolescence on manifestation of aggression. Third, we examine recent data linking use of synthetic cannabinoids (SCs) to manifestation of aggressive behavior. Fourth, we discuss violence arising from cannabis use in schizophrenia (SCZ) and other common psychiatry disorders. Fifth, we highlight the available evidence pointing to alterations of the ECS in borderline personality disorder (BPD) and antisocial personality disorder (ASPD), two psychiatric conditions characterized by high levels of violence.

## ECS and aggression in animal models

While a full discussion of the mechanisms and actions of the ECS is beyond the scope of this review, we provide a brief overview. Stimulated by Δ9-tetrahydrocannabinol (THC), the primary psychoactive ingredient in cannabis, the ECS modulates the activity of a large number of brain neurotransmitters and is a potent modulator of myriad neural circuits influencing human behavior (Basavarajappa, 2007; Crowe et al., 2014). Endogenous cannabinoid ligands (endocannabinoids) are fatty acid amides and monoacylglycerols that function as lipid neuromodulators. Unlike most other neurotransmitters that are stored in vesicles, endocannabinoids exhibit rapid, on-demand synthesis in response to neuronal activation, and once synthesized, undergo retrograde synaptic transmission to the extracellular space where they bind to presynaptic endocannabinoid receptors (Basavarajappa, 2007; Crowe et al., 2014). This style of neurotransmission is known to precisely regulate information flow within most major neurotransmitter pathways and contributes to the synaptic plasticity of brain regions involved in syndromes associated with violent behavior (Basavarajappa, 2007).

Several investigations (Supplementary Table 1, Sheet 1) have documented an anti-aggressive effect of THC in animals (Dorr and Steinberg, 1976; Miczek, 1978). THC binds to cannabinoid CB1 receptors in the central nervous system to exert its psychoactive effects (Matsuda et al., 1990). In a landmark investigation (Miczek, 1978), low-dose THC was administered to resident mice, rats, and squirrel monkeys prior to their participation in a resident-intruder paradigm. Results revealed a decreased frequency of attacks by resident animals in a dose-dependent manner. That is, higher doses of THC were associated with lower attack frequencies. THC was injected intraperitoneally in mice and rats and delivered orally to monkeys. THC administration to intruder mice, rats, and squirrel monkeys did not change defense, submission, or flight reactions when these animals were paired with non-treated attacking resident opponents. Doses ranged from 0.125 to 4.0 mg/kg of THC. Another study (Van Ree et al., 1984) examined social contact behavior in isolated rats that had received low or high doses of THC injected intraperitoneally or cannabidiol, a phytocannabinoid derived from cannabis that is not an addictive drug but may have anxiolytic effects (Crippa et al., 2011). While higher doses of THC (10 mg/kg) had a suppressive effect on social interactions, lower doses (1 mg/kg) decreased aggressive behavior, including fighting, kicking, or biting. Cannabidiol had no effect on social contact behaviors. An investigation conducted in pigeons similarly reported a negative correlation between an injected THC dose (0.5 mg/kg or 1.0 mg/kg) and aggressive responding (Cherek et al., 1980).

On the other hand, some animal studies have failed to detect an effect of THC on aggression [(Sieber et al., 1980) (20 mg THC/kg administered orally), (Cutler and Mackintosh, 1975) (5 mg/kg administered by intraperitoneal injection)] or, alternatively, have reported increased aggressive behavior following cannabinoid or THC administration. On the other hand, providing aggressive, electrically shocked rats with propylene glycol and marijuana (1 mg THC/kg ingested orally) increased aggressive responding (Carder and Olson, 1972). A subsequent investigation (Ueki, 1979) reported that group-housed rats became significantly more aggressive—fighting between cage mates and muricide emerged—by chronic daily doses of THC (6 mg/kg injected intraperitoneally). Aggression manifested approximately two weeks into treatment. Thereafter, even a single dose of THC elicited an attack response or muricide if rats were isolated. Aggressive behavior was maintained as long as rats were held in isolation. Once transferred to group housing, however, muricide decreased by 50% and attacks were reduced. Why does THC/cannabis administration appear to provoke aggression in some settings but not others? Several possibilities can reconcile these apparent discrepancies. One, the dose and delivery of administered THC appear to be important variables. In general, studies that used smaller doses of THC/cannabis were less likely to report the emergence of aggression. In some instances, aggression even decreased. Two, aggressive responding may be related to the chronicity of THC exposure in animals. Three, adverse environmental manipulations could also impact aggressive behavior when combined with THC intake. Four, the rearing environment, namely whether animals are housed in isolation, as a group, or transferred from one setting to another, may impact tendency toward aggressive responding. One could also speculate that THC withdrawal as opposed to THC administration may increase aggression. It is important to note, however, that these animal results do not necessarily translate to humans, given the complex interplay of biopsychosocial factors that can precipitate aggression in *homo sapiens*.

Since CB1 receptors are the most abundantly expressed G protein-coupled receptors in the central nervous system (Herkenham et al., 1990) and transduce signals upon binding to THC, research examining this component of the ECS and its relation with aggression could yield important information. CB1 receptors are located on serotonergic, noradrenergic, dopaminergic, GABAergic, and glutamatergic nerve terminals (Hermann et al., 2002; Häring et al., 2007; Oropeza et al., 2007; Azad et al., 2008; Kano et al., 2009; Morozov et al., 2009), with signaling effects most prominent at GABAergic and glutamatergic synapses (Katona and Freund, 2012). It is worth noting that dose-dependent effects of THC and cannabinoids in aggression are likely linked to the fact that CB1 agonists at low doses increase serotonin (5-HT), while at lower doses induce an abrupt decrease of 5-HT (Bambico et al., 2007). One study employing the resident-intruder paradigm compared CB1 knockout (CB1KO) mice with wild-type. Results indicated that mice devoid of CB1 receptors were more aggressive toward intruders than wild-type but only during the first testing session (Martin et al., 2002). In a subsequent study that analyzed social encounters with conspecifics, group-housed CB1KO mice, when compared with wild-type, were found to spend more time in threat and attack behaviors, exhibit aggressive behavior sooner, and engage in longer periods of aggression during a social interactions test (Rodriguez-Arias et al., 2013). Interestingly, administering the CB1 agonist arachidonyl-2′-chloroethylamide (2 mg/kg injected intraperitoneally) to single-housed aggressive mice decreased aggression. These results highlight the importance of CB1 neurotransmission as a potential anti-aggressive signaling pathway.

A subsequent investigation looked at whether cannabinoid CB2 receptor knockout (CB2KO) mice similarly displayed increased aggression compared with wild-type during the social interaction test and resident-intruder paradigm (Rodriguez-Arias et al., 2015). CB2 receptors are mainly localized to immune cells (Pertwee, 2005) but have also been detected in several areas of the rat brain, including cerebral cortex, striatum, amygdala, thalamus, cerebellum, spinal nucleus, olfactory nucleus, and hippocampus (Gong et al., 2006; Onaivi et al., 2008). It has been suggested that CB2 receptors can only be measured in the brain during situations of neuroinflammation (Benito et al., 2008). Study results (Rodriguez-Arias et al., 2015) indicated that group-housed CB2KO mice devoted more time to threat and attack behaviors, engaged in threat and attack activities for longer periods, and launched more attacks than wild-type group-housed mice. Increased aggression manifested during both tasks. The authors also reported that acute administration of a CB2 agonist (1, 2, and 4 mg/kg of JWH133 injected intraperitoneally) to isolated Oncins France 1 (OF1) mice—a strain that had been selectively bred for aggressive behavior—decreased aggression. However, pre-treatment of OF1 mice with a CB2 antagonist (2 or 4 mg/kg of AM630 injected intraperitoneally) and then application of JWH133 (2 or 4 mg/kg) resulted in animals spending more time attacking than mice treated with the CB2 agonist alone. The results of these CB1 and CB2 receptor KO studies suggest that decreased activation of this receptor system may be linked to emergence of aggressive behavior in certain animals. Conversely, stimulation of CB1 and CB2 receptors appears to exert pacifying effects.

One of two major endogenous ligands and primary molecular targets of CB1 receptors is anandamide (AEA). AEA is synthesized on-demand in postsynaptic membranes (Kano et al., 2009) and then feedbacks in retrograde fashion onto presynaptic CB1 receptors, whose activation inhibits afferent neurotransmitter release (Ohno-Shosaku and Kano, 2014). Similar to studies reporting a biphasic effect of THC on aggression, there is some evidence that AEA influences aggressive behavior in a dose dependent manner (Sulcova et al., 1998). In one model of agonistic behavior, singly housed mice were divided into two groups based on whether they attacked opponents (e.g., aggressive mice) or exhibited defensive-escape behavior without any attacks (e.g., timid mice). Principal study findings included the observation that lower doses of AEA (0.01–0.1 mg/kg) administered systemically did not affect agonistic behavior in aggressive mice. However, the highest dose tested (10 mg/kg) significantly reduced aggression in aggressive mice, while stimulating timidity in aggressive mice. On the contrary, the lowest dose of AEA (0.01 mg/kg) roused aggressive behavior in timid mice, while the highest dose (10 mg/kg) had no effect on aggressive behavior. In addition to dose-related effects, these results point to possible predisposing factors, such as temperament, that may mediate expression of aggression through endocannabinoid signaling.

2-arachidonoylglycerol (2-AG) is another endogenous ligand of cannabinoid receptors with similar properties as AEA (Morena et al., 2016). 2-AG is metabolized by the hydrolytic enzyme monoacylglycerol lipase (MAGL), which is located near CB1 receptors in presynaptic terminals (Gulyas et al., 2004). The impact of 2-AG neurotransmission on aggressive behavior was evaluated in a recent study that employed both a MAGL inhibitor (JZL184) and CB1 receptor antagonist (AM251). MAGL inhibition (JZL184; 8 and 16 mg/kg injected intraperitoneally) was shown to reduce the number of bites delivered and increase the amount of bites received by resident CD1 mice during the resident-intruder paradigm. At higher doses of the MAGL inhibitor (16 mg/kg), mice received more bites than they initiated, while level of defensiveness was unchanged. Adding a CB1 receptor antagonist (AM251; 0.5 mg/kg injected intraperitoneally) did not dampen the effects of MAGL inhibition, suggesting that the results achieved by administering a MAGL inhibitor were not mediated through CB1 receptors (Aliczki et al., 2015). Among intruders treated with the MAGL inhibitor, more bites were received than delivered and mice also engaged in greater defensive behavior versus offensive strategies. The authors noted that previous findings describing a link between cannabinoids and aggression were largely dependent on the testing conditions (e.g., stressfulness of experimental manipulation, timing of testing, and duration of treatment) and were generally low in magnitude. By contrast, MAGL inhibition differed from other cannabinoid treatments, as both biting and offensive behavior were abolished in treated mice. Notably, these observations were present in both residents and intruders, which occupied different hierarchical positions in the paradigm employed. Replicating these results in the same and other species by employing a variety of experimental manipulations would be important next steps to establishing MAGL inhibitors as potent negative modulators of aggression.

The ECS has also been probed in male Syrian hamsters (Moise et al., 2008). The study in question investigated the effect of CB1 receptor blockade (rimonabant 5 mg/kg injected intraperitoneally or AM251 5 mg/kg injected intraperitoneally) and fatty acid amide hydrolase (FAAH) inhibition (URB597; 0.3 or 3 mg/kg injected intraperitoneally) on a variety of paradigms, including models of conditioned and unconditioned social defeat. FAAH is a membrane-derived lipid modulator that is detectable on intracellular membranes in postsynaptic neurons. The enzyme degrades AEA, presumably leading to decreased AEA-induced CB1 neurotransmission. Although conditioned and unconditioned social defeat paradigms are not specifically designed to assay aggression in the same way as the resident-intruder paradigm, the results obtained can inform on behavior that may be compatible or incompatible with aggressive responding. In this experiment, acquisition of unconditioned social defeat was achieved by re-locating an experimental hamster to the home cage of a known resident intruder for 15 min on day 1. Experimental animals exhibited submissive and defensive behavior toward the resident aggressor. Conditioned defeat was achieved 24 h after the initial acquisition of unconditioned defeat in experimental hamsters. A non-aggressive intruder was then transported to the home cage of the experimental hamster for 5 min on day 2. This interval enabled an adequate sampling of behaviors and ensured that behaviors were consistent between tests. For example, defeated hamsters in these models typically display defensive behavior, circumvent social encounters, and exhibit a lack of natural territorial aggression (Jasnow et al., 2005). Study results in the present investigation confirmed that experimental hamsters exhibited unconditioned social defeat on day 1 when exposed to a dominant hamster; that is, they displayed heightened submissive/defensive behavior compared with any other category of behavior. On day 2, they subsequently manifested conditioned defeat, or behavior similar to unconditioned social defeat, upon exposure to smaller, non-aggressive stimulus hamsters. Although diazepam (2 or 6 mg/kg administered intraperitoneally), a benzodiazepine, decreased submissive and defensive behavior during conditioned defeat, aggressive behavior did not differ by dose. Neither FAAH nor CB1 receptor blockade altered expression of conditioned defeat. Furthermore, none of these pharmacologic manipulations transformed behavior acquired on day 1 during the unconditioned social defeat model. Future studies examining the potential role of FAAH in relation to aggressive behavior should involve paradigms especially designed to measure indices of aggression (e.g., resident-intruder aggression assay) in addition to investigating other species.

In summary, although many studies have reported reduced aggression following THC administration, others have reported opposite effects. This discrepancy could be related to dose, chronicity of exposure, or concurrent environmental manipulations. CB1 agonism appears to exert anti-aggressive effects, while CB1KO models are associated with increased aggression. Effects regarding CB2 receptors appear similar: CB2KOs manifest heightened aggression, while agonists at the CB2 receptor lessen aggression. AEA exerts biphasic effects on aggression, which may depend on the temperament of the animal. MAGL inhibition appears to reduce aggression in mice and at higher levels makes them more vulnerable to attack. Results of a conditioned and unconditioned social defeat paradigm in hamsters were unaffected by FAAH administration. However, future studies could discover a role for FAAH in paradigms specifically designed to elicit aggressive responding.

## Developmental effects of cannabis exposure

The effect of cannabis exposure during prenatal and perinatal periods as well as in adolescence has been studied in humans (Supplementary Table 1, Sheet 2). Among prenatally cannabis-exposed males and females, girls, but not boys, scored higher on measures of aggression and inattention at age 18 months, although these effects were no longer present at 36 months (El Marroun et al., 2011). Exposure to cannabis during gestation has also been correlated with altered scores on the Self Report Delinquency Scale (Loeber et al., 1989), which includes violence as a subscale, during adolescence (Day et al., 2011). Some research has also found that attention deficits present by age six years can intensify to delinquency and externalizing behaviors in children with prenatal exposure to cannabis (Goldschmidt et al., 2000). Regarding exposure to cannabis in adolescence, preliminary findings indicate that use of cannabis is associated with property and violent crime, especially during the ages of 14–15 years (Fergusson et al., 2002). While these studies provide initial evidence of a link between cannabis exposure at different developmental windows and aggression, more research is required to mechanistically discern how exposure at different time points can lead to aggressive outcomes.

## SCs and aggression

SCs are a heterogeneous group of compounds that are sold as herbal matter to be smoked or consumed in other ways. SCs share some properties in common with THC but also show important differences. They are highly lipophilic and cross the blood-brain-barrier easily (Dhawan et al., 2006). In contrast to THC that has weak partial agonist activity at the CB1 receptor, most SCs exhibit full CB1 receptor agonist activity (Elsohly et al., 2014). Moreover, THC displays only modest affinity for the CB1 receptor, whereas SCs show higher affinity and intrinsic activity at this same receptor. Thus, SCs exert stronger agonist action at the CB1 receptor in terms of efficacy (Hillard et al., 2012; Van Amsterdam et al., 2015). As SCs do not contain cannabidiol or cannabivarin, they may lack some of the intrinsic antipsychotic and anxiolytic properties of natural cannabis (Iseger and Bossong, 2015), although it is not universally accepted that cannabidiol or cannabivarin have antipsychotic or anti-anxiety effects. Furthermore, since there is no standardization of SC products, the concentration of active ingredients can vary significantly within batches (Sedefov et al., 2009; Vandrey et al., 2012). Consequently, the clinical effects of SCs can be highly unpredictable, even among people who have smoked the same batch together (Kronstrand et al., 2013). Acute effects of SCs can include a wide variety of symptoms, some of which may resemble cannabis intoxication. Characteristic symptoms of SC not seen in cannabis intoxication include agitation, seizures, hypertension, emesis, and hypokalemia. SC consumption often presents with a constellation of psychiatric symptoms, including agitation, anxiety, irritability, hallucinations, cognitive impairment, and psychosis (Brents et al., 2011; Hurst et al., 2011; Hermanns-Clausen et al., 2013; Fattore, 2016). Repeated use of SC can induce tolerance, while discontinuation after prolonged use can lead to withdrawal symptoms (Vandrey et al., 2012). Very little is known about the long-term sequelae of chronic SC use, although self-harm, including self-inflicted burns, have been reported (Meijer et al., 2014).

Case reports have chronicled aggressive behavior accompanied by delirium, anxiety, and psychosis in people with no previous medical history following consumption of SCs (Schwartz et al., 2015). The higher incidence of psychotic symptoms among SC users, including agitation and aggression, suggests that the active ingredients contained within SCs may affect the neural pathways implicated in the manifestation of psychotic symptoms. In a chart review of patients who had been admitted to a dual disorders psychiatric unit, individuals who used both THC and SCs were rated as more aggressive than patients using SCs alone, THC alone, or neither, while those using SCs but not THC had the highest levels of agitation (Bassir Nia et al., 2016). A final study compared risk taking and violent behaviors among youth using SCs and cannabis in a nationally representative sample of students from grades 9–12. The investigation found that those who had ever used SCs, compared with students who had only experimented with THC, were more likely to have engaged in sexual violence during dating, physical violence during dating, forced sexual intercourse upon another individual, injuring someone with a weapon on school property, physical fights, and carrying a weapon (Clayton et al., 2017). However, these results remain silent on whether use of SCs leads to aggression and agitation or if youth with pre-established aggressive tendencies are more prone to experiment with SCs. Clearly, the effects of SCs will depend on their constituent components, which differ between the various SCs.

## Cannabis, other psychiatric presentations, and aggression

Use of cannabis has also been implicated in the violence of other psychiatric conditions. Here, we provide brief highlights of these relationships. For example, in a sample composed mainly of patients with affective disorders who had been recently discharged from hospital, persistent use of cannabis (cannabis use was coded dichotomously) was associated with violent behavior at several time points (Dugré et al., 2017). The combined use of cannabis (no specific amounts reported) and alcohol was also found to predict violence in SCZ (Koen et al., 2004). Another population study reported an odds ratio of over 18 for cannabis dependence (average amount used not reported) and comorbid SCZ-spectrum disorder with violent offending (Arsenault et al., 2000). However, not all investigations have reported connections between cannabis use and violence in SCZ (Arango et al., 1999). Heterogeneity of results is likely due to variable amounts of cannabis ingested between groups; whether a patient is an inpatient vs. outpatient, thus affecting his or her ability to use; and overall frequency of use.

## BPD, violence, and the ECS

BPD is a debilitating psychiatric condition that affects 2% of the general population, 10% of psychiatric inpatients, and 20% of psychiatric outpatients (Widiger and Weissman, 1991; Torgersen et al., 2001). Core symptom clusters of BPD include extreme dysphoric moods; self-destructive, impulsive behavior; and disinhibited anger (Leichsenring et al., 2011). These symptoms drive the recurrent, self-directed aggression and suicidal behavior that account for the exceedingly high morbidity and mortality of BPD (Leichsenring et al., 2011). The gravity of self-directed violence in BPD is reflected by several disturbing statistics: over 60% of BPD patients self-mutilate (e.g., cutting wrists or burning skin), 60–70% of patients with BPD attempt suicide, and 8–10% successfully commit suicide (Soloff et al., 1994; Gunderson, 2001; Gunderson and Ridolfi, 2001; Oldham, 2006).

One report found that serum levels of AEA were higher in BPD (Schaefer et al., 2014). Whole blood samples were obtained from 26 patients with BPD, some of whom had comorbid posttraumatic stress disorder (PTSD); 21 individuals with PTSD only; and 30 healthy controls. However, members from each group were using cannabis and a significant proportion of BPD and PTSD subjects were taking psychotropic medications. Elevated serum levels of AEA were found in BPD compared with the two control groups. Overall, the relationship between plasma ECs and brain function is difficult to interpret (Hillard, 2018). A subsequent study found that AEA was reduced in the cerebrospinal fluid of BPD (Koethe et al., 2014). These subjects were likely taking medications and using illicit substances, making it difficult to reconcile differences in findings between plasma and CSF ECs among BPD patients.

## ASPD, violence, and the ECS

ASPD is a chronic mental condition that affects 1% of American adults (Lenzenweger et al., 2007). Individuals with ASPD consistently engage in reckless, irresponsible, and impulsive behavior from youth onward, and the interpersonal style of ASPD is characterized by manipulation, deceitfulness, and a callous disregard for the rights of others (Ogloff, 2006). Half of all people with ASPD possess a record of criminal offending, and 85% have a history of violent behavior toward others (Robins and Regier, 1991; Samuels et al., 2004). ASPD is associated with the highest rate of violence toward children, intimate partners, and strangers among all psychiatric disorders (Coid et al., 2006). Unsurprisingly, approximately 50% of incarcerated individuals meet diagnostic criteria for ASPD (Fazel and Danesh, 2002). In addition to violence toward others, ASPD is also associated with increased risk of death by violent suicide (Repo-Tiihonen et al., 2001).

A single genetic association study of 137 alcoholic males found that a polymorphism of the gene coding for FAAH (C385A) was associated with a diagnosis of ASPD. The A/A genotype of the FAAH gene is associated with decreased FAAH expression and activity in humans (Chiang et al., 2004). Multivariate regression analysis additionally revealed that higher impulsive-antisocial psychopathic traits predicted the C/C FAAH genotype (Hoenicka et al., 2007), or greater *in vitro* levels of FAAH. The A/C and A/A genotypes similarly result in greater AEA (Sipe et al., 2002). Finally, a positron emission tomography study of CB1 receptor availability in 47 healthy individuals reported that novelty seeking, a core feature of ASPD that is highly correlated with aggression (Raine et al., 1998), was inversely correlated with CB1 receptor availability, especially in the amygdala (Van Laere et al., 2009). While these results were obtained in a non-clinical sample, they could shed light on the molecular underpinnings of pathological personality traits in ASPD. Furthermore, there is consistency between lower *in vitro* levels of AEA in ASPD reported in the study by Hoenicka et al. (2007) and increased CB1 receptor availability among individuals with high antisocial traits. Quantifying brain levels of AEA and CB1 receptor availability in ASPD would be a crucial next step to test this mechanism. Importantly, the mechanism between self-harm and aggression toward others shares similarities (Chester et al., 2015).

## Conclusion

This review has explored the bench to bedside work highlighting the role of endogenous and exogenous cannabinoids in relation to aggressive behavior. By far, the largest corpus of evidence originates from preclinical models that have examined the major components of the ECS and aggression. With respect to SCs, their unique pharmacodynamic properties differ from those of THC, which perhaps accounts for discrepancies in side effect profiles, including enhanced aggression and agitation in SCs. Finally, preliminary evidence links ECS abnormalities to aggressive psychiatric conditions, such as BPD and ASPD. The next challenge will be to draw connections between indices of aggressive and violence in these disorders with alterations of the ECS. In conclusion, continued exploration of the highly nuanced ECS will only bolster our understanding of human aggression and violence.

## Author contributions

NK and AM were both responsible for researching and writing the article.

### Conflict of interest statement

The authors declare that the research was conducted in the absence of any commercial or financial relationships that could be construed as a potential conflict of interest.
